# Inhalational Drug Devices: Revisiting the Linchpin of Asthma Management

**DOI:** 10.3390/jpm14080867

**Published:** 2024-08-16

**Authors:** Damini Saxena, Neveda Murugesan, Uyioghosa D. Evbayiro, Marina K. Ngassa, Muhammad Adrish

**Affiliations:** 1 Baylor College of Medicine, Section of Pulmonary, Critical Care, and Sleep Medicine, 1 Baylor Plz, Houston, TX 77030, USA; neveda.murugesan@bcm.edu (N.M.); muhammad.adrish@bcm.edu (M.A.); 2 Baylor College of Medicine, Section of General Internal Medicine, 1 Baylor Plz, Houston, TX 77030, USA; uyioghosa.evbayiro@bcm.edu (U.D.E.); marina.ngassa@bcm.edu (M.K.N.)

**Keywords:** asthma, inhalers, management, patient education

## Abstract

Asthma remains a prevalent condition among all age groups globally. First-line treatment requires the delivery of medications into the distal respiratory tract via inhalers. Using appropriate inhaler techniques is a significant challenge in achieving disease control. A variety of inhalers are available for treating asthma, and selecting the appropriate inhaler type for any given patient is crucial to achieving and maintaining symptomatic control. This review will discuss the anatomy and physiology behind drug delivery via inhalers, the types of inhalers currently available for use, nebulizers, and future directions in the delivery of inhaled medications for asthma.

## 1. Introduction

Obstructive lung diseases, such as asthma, remain prevalent among children and adults within the United States and globally. Asthma affects almost 25 million people in the United States alone [1]. Although often considered a pediatric illness, five times as many adults are living with asthma than children [1]. Uncontrolled asthma results in significant morbidity and mortality, claiming over 10 million deaths in 2021 alone [1,2].

Inhalers remain the mainstay of therapy as per the Global Initiative for Asthma (GINA) 2023 document [3]. Extensive coaching to the patient and feedback from the provider on appropriate inhaler techniques are necessary to ensure adequate delivery of medications. Yet, this remains one of the greatest barriers to achieving disease remission in asthma patients, leading to more asthma exacerbations requiring oral corticosteroids and antibiotics, emergency room visits, and hospitalizations [4,5]. Inhaler misuse increases the most with older age and with lack of adequate instruction from healthcare providers [5]. The onus lies with healthcare providers in selecting optimal inhalers for patients and providing patient education.

A variety of inhalers and nebulizers have been developed over the years to counter this issue. The use of inhalational therapies dates back many centuries across myriad cultures, while the first pressurized metered dose inhaler was developed in 1956 by Riker Laboratories Inc. (now 3 M Delivery Systems™) [6]. Many more inhalational delivery systems have been innovated since then. Knowing the different types of inhalational delivery systems and selecting the appropriate device for any given patient with asthma may be crucial in achieving adequate disease control [3].

## 2. Anatomy and Physiology of the Respiratory Tract

The respiratory tract is divided by anatomic location (upper and lower respiratory tracts) and physiologic zone (conducting and respiratory zones). The upper respiratory tract consists of the nasopharynx and larynx lined by a lymphoid and mucoid barrier. The lower respiratory tract consists of the trachea, bronchi, respiratory bronchioles, and alveoli. It is lined by columnar epithelia with lymphoid cells, goblet cells that secrete mucus to trap foreign matter, and cilia, which sweep mucus towards the upper respiratory tract for elimination [7]. The lower respiratory tract is also surrounded by smooth muscle cells, which regulate the diameter of the airway in response to cholinergic activity by the vagus nerve [8]. The conducting zone, which warms and humidifies air as it passes through the respiratory tract, includes the upper respiratory tract to the terminal bronchioles. The terminal bronchioles to the alveolar sacs are the respiratory zone and the sites of gas exchange. 

**Figure 1:**
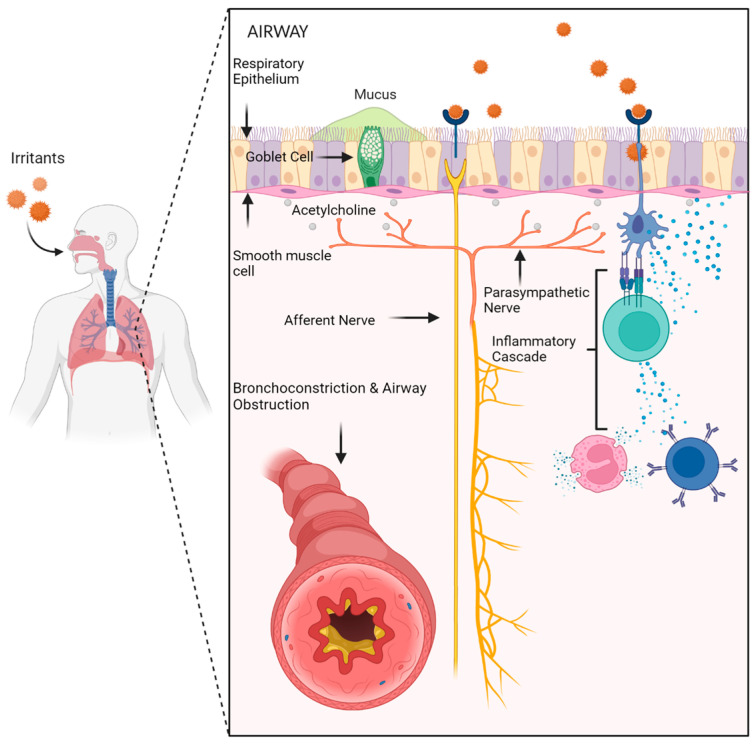
Asthma cascade: Inhalation of irritants activates inflammatory cells and induces parasympathetic response via the vagus nerve. Acetylcholine is released at muscarinic receptors, inducing smooth muscle constriction and increased mucus production by goblet cells. Inflammatory cells release cytokines that potentiate the parasympathetic response, resulting in bronchoconstriction and airway obstruction. Created on Biorender.com.

In asthma, an environmental allergen activates respiratory receptors, resulting in a parasympathetic response via vagal stimulation (Figure 1). Acetylcholine is bound to acetylcholine receptors on goblet cells and smooth muscle cells, leading to increased mucus secretion and decreased airway diameters, respectively, obstructing airflow and limiting gas exchange [8]. This is further compounded by the activation of lymphoid cells, which concurrently release cytokines that compound smooth muscle cells’ constriction and airflow obstruction.

**Figure 2:**
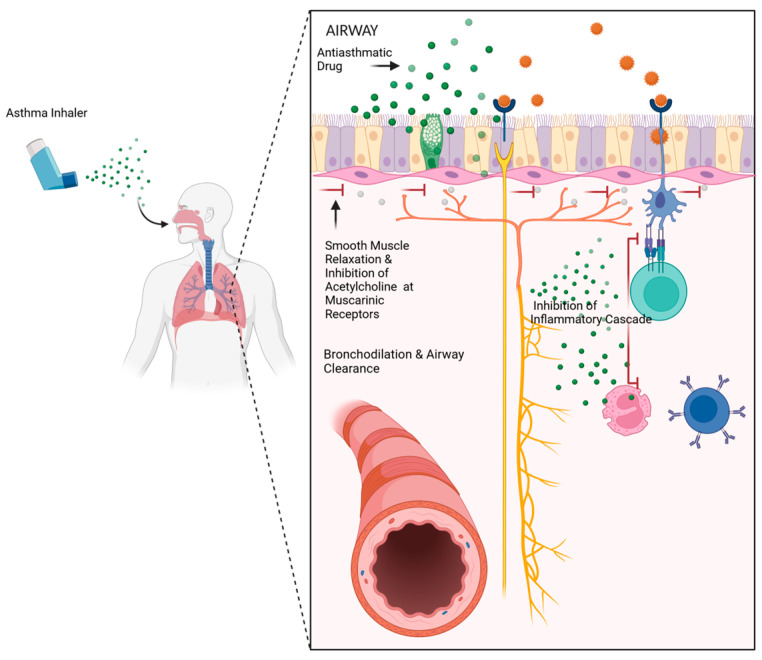
Inhaler drug delivery: Inhalation and sedimentation of anti-asthma medications in distal branches of the respiratory tree relax smooth muscles via β_2_ receptors (β_2_-agonists), inhibit acetylcholine action at muscarinic receptors (antimuscarinics), and inhibit inflammatory cells and cytokine release (glucocorticoids). This subsequently induces bronchodilation and relieves airflow obstruction. Created on Biorender.com.

Asthma inhalers work through different mechanisms to improve gas exchange in the lungs and are grouped by the level of the inflammatory cascade they act on (Figure 2). Bronchodilators work through the relaxation of bronchiole smooth muscle, allowing bronchial dilation, improved airflow, and effective gas exchange. They are further subclassified into β_2_-agonists, which relax bronchial smooth muscles through direct action on β_2_ receptors, and muscarinic antagonists, which inhibit cholinergic induction of bronchoconstriction triggered by environmental allergens. Inhaled corticosteroids (ICSs) exert their anti-asthmatic effects by inhibiting lymphoid cells involved in the inflammatory cascade and work in synergy with bronchodilators, upregulating β_2_ receptors and enhancing bronchodilatory effects [9]. Finally, methylxanthines are a class of medications used in asthma that exert bronchodilatory effects through adenosine receptor antagonism and phosphodiesterase-3 (PDE-3) receptor inhibition [10].

Asthma inhalers are manufactured in various inhaler devices with unique delivery mechanisms for effective delivery into the lungs. These include pressurized meter dose inhalers (pMDIs), dry powder inhalers (DPIs), soft mist inhalers (SMIs), and nebulizers. The efficacy of each asthma inhaler device is driven by the amount of drug deposited into the distal lungs. This is impacted by several factors, notably the particle size of the drug, inhaler technique, and accessory devices available for each inhaler type. When inhaled, most particles with a size of 10 µm or larger are deposited in the upper airways due to the impaction of these larger molecules along the walls of the respiratory tract [11]. Smaller particles ranging from 0.5 µm to 5 µm remain suspended along the respiratory tract before depositing along the distal airways and alveoli through sedimentation. Below is a brief description of currently available inhalational devices in asthma.

## 3. Pressurized Metered Dose Inhalers

pMDIs are the most used reliever inhaler devices, with a global prevalence of approximately 97% [12]. They are used to administer β_2_-agonists, antimuscarinic agents, and ICSs (Table 1).

**Figure 3:**
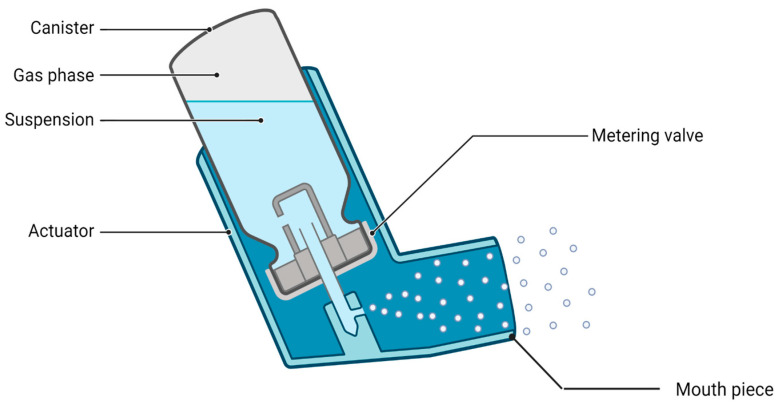
Schematic of a pMDI device. Created on Biorender.com.

pMDIs are comprised of a pressurized canister, an asthma drug in a solution or suspension, a metering valve that controls the delivery of drug particles measuring from 2 µm to 4 μm in size, and a mouthpiece actuator (Figure 3). The drug being administered is intermixed with a propellant (typically hydrofluoroalkane, or HFA), which helps to actively propel the drug from the device into the airway [13]. pMDIs are portable, cost-effective, and generally able to deliver a consistent amount of propelled drug particles that can reach the distal airways.

Despite these advantages, pMDIs have their shortcomings primarily due to ineffective drug delivery arising from a poor inhaler technique. The proper closed mouth technique with pMDIs involves full exhalation, then activating the pMDI while simultaneously inhaling slowly and deeply, with a 10 s breath hold at the end of inhalation to allow for sedimentation of drug particles in the airways [14]. Frequent priming of the device before use is also needed. In a systematic review of errors in inhaler device therapy for obstructive lung disease, pMDIs had the highest frequency of errors, with over 40% of patients demonstrating an inappropriate inhaler technique [14]. The most frequent errors involved poor coordination between actuation and inhalation, depth of inhalation, and breath-holding at the end of inhalation [14]. Even with an adequate technique, pMDIs are limited by inefficient drug delivery, with only half of the dispensed drug reaching the distal airways and the rest depositing in the oropharynx and upper airways [15].

Due to the challenges associated with pMDI use, there is a concerted effort to improve patient education on the proper technique, alongside the development of new pMDI device types, such as breath-actuated MDIs (BA-MDIs), and accessory devices to help simplify the inhalation process and improve drug delivery to the lungs. Accessory pMDI devices include spacers, which are extended tubing attached to the mouthpiece of pMDIs, and valve-holding chambers, which are extended tubing with a one-way valve. Both BA-MDIs and spacers serve as a reservoir for the aerosolized drug particle, decreasing the aerosol’s speed and particle size, effectively reducing drug deposition in the upper airway, and improving drug delivery into the lungs [16]. BA-MDIs are comprised of a pressurized canister and a trigger mechanism released by a spring during inhalation [17]. This mechanism of action alleviates inhaler difficulties for patients unable to coordinate actuation with inhalation of pMDIs and requires no priming. Redihaler^®^ is a common BA-MDI used in the United States, and it is usually paired with beclomethasone, an ICS, for the treatment of asthma.

## 4. Dry Powder Inhalers

Traditional asthma treatments, including bronchodilators, steroids, mast cell stabilizers, and anticholinergic drugs, have mainly relied on the pMDI. However, pMDIs are inefficient at delivering drugs to the distal airways and are rife with patient-centric challenges, as noted above. This method is, furthermore, facing scrutiny due to environmental concerns about the use of chlorofluorocarbon (CFC) propellants [18]. DPIs are newer inhalers that do not have propellants. Medications in DPIs are stored as a fine powder, which is more stable in storage compared to the liquid formulations in pMDIs [19]. They are designed for ease of use, featuring a preloaded dose mechanism that eliminates the need for coordination between actuation and inhalation [19]. Patients simply inhale through the mouthpiece, which triggers the release of a precise dose of medication.

FDA-approved DPIs include one to two classes of medications and may be used once daily or up to four times daily. All DPIs include ICSs, β_2_-agonists, antimuscarinics, or a combination of these classes (Table 2).

The clinical efficacy of DPIs is influenced by three main factors: the formulation type, the device used, and patient factors. Key considerations for formulations include the drug’s physiochemical properties, such as solubility, particle size, morphology, and preparation method [20]. Particles smaller than 0.5 µm may be exhaled immediately or rapidly absorbed systemically, while particles larger than 5 µm may predominantly deposit in the oropharynx. Effective drug deposition relies on a high fine particle dose, fine particle fraction, and low mass median aerodynamic diameter [20]. A drug particle with an aerodynamic diameter of 1–5 µm is most likely to reach the distal airways [19]. The drug formulation is also critical in achieving adequate drug delivery. DPIs using a carrier-free formulation contain only fine particles of the drug and are more likely to aggregate, causing poor flow and nonuniform deposition of medication. DPIs with a carrier formulation, such as fine drug particles mixed with lactose, have improved flowability but poor separability of the drug from the carrier and deposition in the lungs [19]. Most manufacturers of DPIs attempt to balance the above parameters to allow for effective delivery of medications to the lower respiratory tract.

Another challenge of DPIs is that they rely on the patient’s inspiratory force to activate drug delivery. DPIs are activated by a forceful inspiration initiated by the patient. Patient factors such as health condition, age, and ability to use the device correctly play crucial roles. Older patients and those with uncontrolled asthma often struggle with DPI use. Devices also differ in internal resistance to airflow, which affects their performance. Thus, prescribers should consider the patient’s peak inspiratory flow and inhalable volume before prescribing DPIs, opting for simpler devices or once-daily dosing for those with physical or cognitive impairments. The following are some FDA-approved DPIs and their common advantages and disadvantages (Table 3).

There are several misconceptions about DPIs, such as the belief that high-resistance devices are ineffective, extra-fine particles improve distal lung deposition, and devices with flow-rate-independent fine particle fractions deliver more consistently [20]. By addressing these misunderstandings and advancing research in respiratory drug delivery, therapeutic outcomes may be optimized with minimal adverse effects.

## 5. Soft Mist Inhalers

Advances in inhaler technology have introduced low-velocity spray devices called SMIs. These multidose, propellant-free inhalers avoid the “ballistic effects” seen with aerosols from pMDIs, thereby minimizing aerosol deposition in the oropharynx. SMIs contain the drug in a liquid form within a closed system, such as a pre-filled syringe or cartridge. When activated, the drug is dispersed as a slow-moving aerosol cloud using mechanical energy. SMIs offer the advantages of both pMDIs and many DPIs, including high drug deposition rates.

SMIs create aerosols with low momentum and small particles, determined by the nozzle design, which influences drug deposition. Studies indicate that SMIs can achieve lung deposition rates exceeding 60%, independent of inspiratory flow rates, thereby reducing oropharyngeal deposition and increasing distal lung delivery [21]. SMIs may be suitable for shear-sensitive formulations, like biologics and lipid-based nanoparticles, with less drug degradation compared to nebulizers. They offer consistent spray contents and are user-friendly, improving patient compliance, particularly for those with low inspiratory forces. These attributes make SMIs a promising choice for inhalation therapies, enhancing their effectiveness and minimizing their side effects.

Differences in breathing patterns, airway diameters, respiratory rates, and lung volumes from infants to elderly patients pose significant challenges for effective drug delivery into distal airways. A study by Kamin et al. showed that younger children (ages 5–8 years) achieved higher drug deposition into distal airways with SMIs than older children (ages 9–12 years) [22]. There are limited data on SMI usage in older patients, and SMIs require hand dexterity and breath coordination, which can be difficult for pediatric and geriatric patients with coordination issues. Proper education on SMI use is essential, as studies indicate only about 30% of patients use SMIs correctly without training [22]. One systematic review and metanalysis found the most common mistakes in SMI use include not exhaling completely away from the device prior to inhaling, inability to sustain a 10 s breath hold on inhalation, lack of taking a slow, deep breath while pressing the SMI dose release button, not holding the inhaler upright, and not turning the base of the SMI towards the arrow until an audible click was observed [23]. Training improves inhalation techniques and drug deposition, highlighting the importance of considering patient-related factors when using SMIs.

### Respimat^®^ Inhaler

The Respimat^®^ inhaler contains tiotropium bromide and olodateral, a long-acting muscarinic antagonist and long-acting ß_2_ agonist, respectively [24]. It is commonly used as a maintenance inhaler in managing chronic obstructive lung disease (COPD) and asthma. One of the standout features of the Respimat inhaler is its ease of use. The device is compact, lightweight, and requires minimal inhalation effort, making it suitable for patients of all ages, including the elderly and those with severe respiratory difficulties. The clear dosage indicator and simple loading mechanism further enhance user convenience.

## 6. Small-Volume Nebulizers

**Figure 4:**
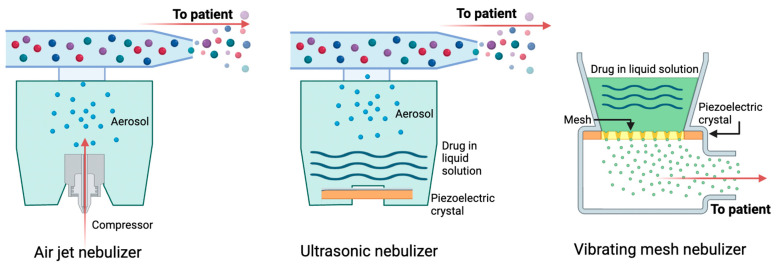
Schematics showing the working principles of the three predominant nebulizer types: air jet nebulizer (**left**), ultrasonic nebulizer (**middle**), and mesh nebulizer (**right**). Created on Biorender.com.

Nebulized medications are additional options for patients to enable the delivery of medications deep into the lungs. Small-volume nebulizers (SVNs) are medical devices that convert liquid medications into a mist for easy inhalation. SVNs are a reliable drug delivery system for those too young or too ill to coordinate and operate other forms of inhalers such as pMDIs and/or DPIs. However, medications cannot be prepared in advance and stored for delivery and aerosolization, but rather must be generated just before inhalation due to their thermodynamic instability. There are three main types of nebulizers: (1) air jet nebulizers, (2) ultrasonic nebulizers, and (3) vibrating mesh nebulizers; a fourth type called smart nebulizers are also in development. Figure 4 shows the schematics of the three most common nebulizers. Table 4 summarizes some of the advantages and disadvantages of all nebulizers.

### 6.1. Air Jet Nebulizers

Air jet nebulizers (AJNs) with corrugated tubing are most common. Compressed gas is funneled through a narrow channel to create a jet of air. The delivery of air through this channel creates negative pressure that draws up drug mixed in liquid. Small droplets of 1–5 µm in size are released, which are easily inhaled. Significant drug loss can occur in exhalation (Table 4).

To combat drug loss during exhalation, AJNs containing collection bags were developed, which release aerosol during inhalation only [25]. The expired air is held in the collection bag and released via a one-way valve. This modification has been shown in studies to aid faster recovery for patients during a bronchospasm [26]. Other modifications to minimize drug loss have led to the design of breath-enhanced (BENs) and breath-actuated AJNs (BANs). BENs use two one-way valves to prevent loss of aerosol into the environment, while BANs can sense inhalation to appropriately regulate the formation and delivery of the medication. The first generation of BANs used thumb control, forcing the patient to block the port that directed gas to the nebulizer during inspiration. This mode required excellent hand–breath coordination and significantly increased the time to complete treatment. Advancements in BANs led to the development of mechanical breath-actuated devices, in which a spring-loaded one-way valve replaces the function of the thumb during inspiration.

Each AJN has a specific flow requirement, which directly correlates with the particle size and drug delivery. Generally, clinicians, respiratory therapists, and patients must understand how to appropriately adjust the flow meter according to the specific AJN. If the compressor does not produce the adequate gas flow, this can lead to an increased particle size and significant drug loss during expiration, resulting in decreased drug efficacy [27].

### 6.2. Ultrasonic Nebulizers

Ultrasonic nebulizers (UNs) harness the energy of piezoelectric crystals to produce aerosols. Vibrating piezoelectric crystals produce sound waves that are transferred to the surface of the liquid, generating a medicated mist that can be inhaled through a mask or mouthpiece [28]. UNs were initially developed to be used for large-volume medications, such as hypertonic saline, but are now produced for small-volume medications needed in asthma as well. Limitations exist to what formulations can be delivered through UNs (Table 4) [29].

### 6.3. Vibrating Mesh Nebulizers

Vibrating mesh nebulizers (VMNs) can be further subdivided into active mesh nebulizers (AMNs) and passive mesh nebulizers (PMNs). AMN machines utilize a net with multiple apertures (usually only a few micrometers in diameter). Electricity through a piezoelectric element generates high-frequency oscillations of the mesh against a liquid solution, generating aerosols. PMNs, in contrast, use an ultrasonic horn to produce vibrations that push the fluid through the mesh to generate the droplets [28]. Several studies have shown that VMNs consistently create uniform droplets small enough to reach the distal lungs, leading to improved medication efficacy [30].

### 6.4. Smart Nebulizers

One nebulizer at the forefront of innovation employs adaptive aerosol delivery in combination with either BAN or VMN technology. When utilizing BANs, the device uses a pressure transducer to sense inspiratory effort. The data are then analyzed by a microprocessor and proprietary computer programs to control the pattern of aerosol generation and calculate and monitor the delivered dose [31].

## 7. Future Directions

As described in this review, the four types of inhalation devices, including pMDIs, DPIs, SMIs, and nebulizers, have different strengths and weaknesses in terms of cost, portability, treatment time, and performance. There is substantial ongoing research and advances are being made in therapeutic aerosol delivery to improve the use and efficacy of medications for patients with asthma. In recent years, the effects of nebulized magnesium sulfate through VMNs were studied in a pediatric population during asthma exacerbations but found to not reduce hospitalizations [32].

### 7.1. Use of Nanoparticles

The effectiveness of DPIs is contingent on a patient’s inspiratory effort to disperse drug aerosols into distal airways, as well as humidity and moisture, which can lead to the aggregation of the powder and subsequent oropharyngeal impaction. The use of nanoparticles or ultrafine particles in DPI formation is investigational but promising. Nanoparticles’ smaller size results in preferential deposition in the distal airways [33]. Nanoparticles can also be incorporated into liposomes, such as with budesonide and fluticasone/salbutamol combinations [34]. The use of nanoparticle technology has the potential to have a broader application in the world of pulmonary pathology.

However, ongoing challenges with maintaining the stability of nanoparticles during manufacturing, providing preservation during storage, and avoiding host mucociliary and macrophage clearance remain [33]. Toxicity remains a consideration, as studies have shown that inhaled nanoparticles containing iridium can cross from the lungs into the systemic circulation with deleterious vascular effects and across the blood–brain barrier [35,36].

### 7.2. Challenges with Developing Inhaled Biologic Agents

The rise of biologic agents in the treatment of asthma has been at the forefront of innovation in the last decade. Their clinical and commercial success can largely be attributed to their high specificity and excellent safety profiles. However, due to their large molecular sizes and high polarity, biologic medications have limited enteral bioavailability, and the majority need to be administered subcutaneously via injections, which can be a barrier amongst patients [37]. Use of biologics through non-invasive delivery mechanisms is still an area of active research, but the possibility of inhaled biologics is an attractive one due to the ease of use and the potential to further limit systemic adverse effects [38].

Biologic agents are highly sensitive to the extremes of temperatures, shear forces, and light exposures [39]. All subcutaneous biologic injections available today require refrigeration for storage. The innate fragility of the formulation poses a challenge to delivering them via inhalation. The process of nebulization can exert higher stress on compounds and lead to protein denaturation. This is especially true of UNs. Similarly, the use of propellants such as CFCs and/or HFAs used in pMDIs can destabilize the peptides in biologic formulations. DPIs may provide a suitable mode for future inhaled biologic agents, since the formulation does not need to be in a liquid state prior to treatment. However, since DPIs depend on inspiratory pressures to ensure adequate drug delivery, the very young, elderly, or severely ill may be excluded, and the dose deposited into the distal lungs may be variable.

The geometry of the airways, humidity, mucociliary clearance, and alveolar macrophages that maintain the microbiome of the lungs further pose barriers to any inhaled formulation [40]. The choice of biologic therapy is often determined by co-morbidities like vasculitis, which would require inhaled biologics to transverse beyond the lung epithelium into the systemic circulation at therapeutic levels.

Toxicity is another major concern. Protein denaturization leading to aggregation has been associated with amyloid diseases and an increased risk of immunogenicity [41]. A phase I study by Burgess and colleagues investigating the safety of a dry powder formulation of anti-interleukin-13 monoclonal antibody fragment for asthma resulted in one subject testing positive for treatment-associated immunogenicity [42].

### 7.3. Phosphodiesterase Inhibitors

**Figure 5:**
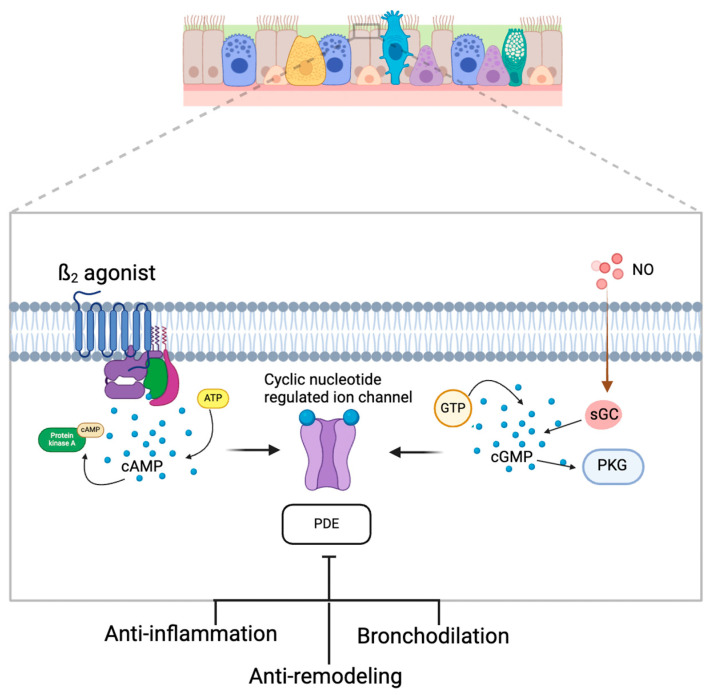
Phosphodiesterase pathways in the airway epithelium and possible inhibitory targets for the treatment of asthma. Created on Biorender.com.

Inhibition of PDE in the airway epithelium as an adjunctive treatment for asthma is no new concept but has been limited by adverse safety profiles during the development and testing of drugs. PDE inhibition prevents the catalysis of cyclic adenosine monophosphate (cAMP) and cyclic guanosine monophosphate (cGMP), leading to prolonged airway smooth muscle relaxation and bronchodilation (Figure 5) [43]. Theophylline was a mainstay in asthma treatment for over fifty years due to its role as a PDE inhibitor but has fallen out of favor due to its narrow therapeutic index and wide range of gastrointestinal, central nervous system, and cardiovascular side effects. PDEs are isozymes that are expressed in a heterogenous manner, and specific PDE subtypes, such as PDE-3 and PDE-5, are predominantly expressed in airway smooth muscle cells [43]. PDE-4 is another potential target given its targetable location on inflammatory cells such as macrophages, eosinophils, and neutrophils [44].

Three PDE-4 inhibitors, namely roflumilast, apremilast, and crisabolore, have been approved for treatment in COPD, psoriatic arthritis, and atopic dermatitis, respectively. There has been a notable gap in the development of PDE inhibitors for the treatment of asthma. There have been several clinical trials investigating the effect of inhaled selective PDE-4 inhibitors, but they all remain in the early stages of research. For example, Singh and colleagues evaluated inhaled CHF6001 in 2016 and noted that while this selective PDE-4 inhibitor significantly reduced late responses to inhaled antigen, there was no statistical difference in the decrease in serum eosinophil count in induced sputum nor in methacholine reactivity post allergen challenge [45].

Research investigating airway inflammation and remodeling in response to inhalation of non-selective PDE inhibitors is also underway. Initial results in mice models appear encouraging, with non-selective PDE inhibitors decreasing many typical features of airway remodeling, including goblet cell metaplasia, mucus hypersecretion, and collagen overproduction and deposition [46]. Ensifentrine, a novel, selective dual inhibitor of PDE-3 and PDE-4, received FDA approval in 2023 for use in patients with moderate to severe COPD, after phase III trials demonstrated significant improvements in lung function, exacerbation rates, and symptom burden [47]. Randomized controlled trials of ensifentrine in asthma are currently underway, showcasing an exciting possibility for new therapeutic options.

## 8. Conclusions

Asthma is a global health condition prevalent across all age groups that is predominantly treated with inhalers. Various types of inhalers have been developed over the last few decades, and new devices are continuously being designed and made available to patients for the treatment of asthma. Selecting the most appropriate inhaler device and ensuring adequate inhaler techniques in each patient is paramount to achieving disease control in the outpatient setting. This review was intended to demystify asthma treatment for the healthcare professional and to spark interest in future directions of respiratory drug delivery devices.

## Figures and Tables

**Table 1:** Classes, medications, and dosing of commonly used FDA-approved pMDIs. Abbreviations: SABA, short-acting ß_2_-agonist; SAMA, short-acting muscarinic antagonist; ICS, inhaled corticosteroid; LABA, long-acting ß_2_-agonist; LAMA, long-acting muscarinic antagonist; HFA, hydrofluoroalkane; mcg, microgram.

Class of Medication	Generic Name	Trade Name	Dose (mcg)
SABA	Albuterol sulfate	ProAir HFA^®^	100
		Proventil HFA^®^	120
		Ventolin HFA^®^	90
	Levalbuterol tartrate	Xopenex HFA^®^	59
SAMA	Ipratropium bromide	Atrovent HFA^®^	17
ICS	Ciclesonide	Alvesco HFA^®^	80, 160
	Mometasone furoate	Asmanex HFA^®^	100, 200
	Fluticasone propionate	Flovent HFA^®^	44, 110, 220
Combination classes: ICS/LABA	Fluticasone propionate/salmeterol xinafoate	Advair HFA^®^	45/21, 115/21, 230/21
	Mometasone furoate/formoterol fumarate dihydrate	Dulera^®^	100/5, 200/5
	Budesonide/formoterol fumarate dihydrate	Symbicort^®^	80/4.5, 160/4.5
Combination classes: LAMA/LABA	Glycopyrrolate/formoterol fumarate	Bevespi Aerosphere^®^	9/4.8
Combination classes: ICS/LABA/LAMA	Budesonide/glycopyrrolate/formoterol fumarate	Breztri Aerosphere^®^	160/9/4.8

**Table 2:** Common FDA-approved classes of medications used in DPIs. Abbreviations: SABA, short-acting ß_2_-agonist; ICS, inhaled corticosteroid; LABA, long-acting ß_2_-agonist; LAMA, long-acting muscarinic antagonist; mcg, microgram.

Class of Medication	Generic Name	Trade Name	Dose (mcg)
ICS	Fluticasone propionate	ArmonAir^®^ Digihaler™ or Respiclick^®^	55, 113, 232
		Flovent^®^ Diskus^®^	50, 100, 250
	Fluticasone furoate	Arnuity^®^ Ellipta^®^	50, 100, 200
	Mometasone furoate	Asmanex^®^ Twisthaler^®^	110, 220
	Budesonide	Pulmicort Flexhaler^®^	90, 180
SABA	Albuterol sulfate	ProAir^®^ Digihaler™ or Respiclick^®^	117
LABA	Salmeterol xinafoate	Serevent Diskus^®^	50
LAMA	Umeclidinium	Incruse^®^ Ellipta^®^	62.5
	Tiotropium bromide	Spiriva^®^ Handihaler^®^	18
	Aclidinium bromide	Tudorza™ Pressair™	400
Combination classes: ICS/LABA	Fluticasone propionate/salmeterol	Advair Diskus^®^ or Wixela™ Inhub™	100/50, 250/50, 500/50
		AirDuo^®^ Digihaler™ or Respiclick^®^	55/14, 113/14, 232/14
	Fluticasone furoate/vilanterol	Breo^®^ Ellipta^®^	100/25, 200/25
Combination classes: LAMA/LABA	Umeclidinium/vilanterol	Anoro^®^ Ellipta^®^	62.5/25
	Aclidinium bromide/formoterol fumarate dihydrate	Duaklir^®^ Pressair^®^	400/12
Combination classes: ICS/LABA/LAMA	Fluticasone furoate/umeclidinium/vilanterol	Trelegy^®^ Ellipta^®^	100/62.5/25, 200/62.5/25

**Table 3:** A list of common FDA-approved DPIs, including the mechanism of use and notable advantages and disadvantages of each DPI. Abbrev.: ICS, inhaled corticosteroid; SABA, short-acting ß_2_-agonist; LABA, long-acting ß_2_-agonist; LAMA, long-acting muscarinic antagonist.

Class of Medication	Generic Name	Trade Name	Inhaler Mechanism	Advantages	Disadvantages
ICS	Budesonide	Flexhaler^®^	Twist the base to load a dose and inhale through the mouthpiece	▪ Dose counter	▪ Dexterity required for twisting
	Mometasone	Twisthaler^®^	Twist the base to load a dose and inhale through the mouthpiece	▪ Dose counter	▪ Dexterity required for twisting
SABA	Albuterol	Respiclick^®^	Pull down cap until audible click and inhale	▪ No priming, shaking, or hand–breath coordination required ▪ Portable ▪ Dose-counter	▪ Costlier than HFA counterparts
LAMA	Aclidinium bromide	Tudorza Pressair^®^	A green control window changes to red with audible click when the dose is correctly inhaled	▪ Visual/audible dose delivery confirmation ▪ Minimal maintenance ▪ Fewer steps to load a dose ▪ Dose preloaded	▪ Costly ▪ Requires twice-daily dosing at fixed 12 h intervals
	Tiotropium	Handihaler^®^	Drug capsule pierced inside a chamber	▪ Requires lower inspiratory force than other LAMA DPIs ▪ Single daily dosing	▪ Cumbersome drug-loading process
ICS/LABA	Fluticasone + salmeterol	Advair Diskus^®^	Manually slide a lever with the thumb, producing an audible click when a dose is ready	▪ Ease of use ▪ Fewer steps to load a dose ▪ Dose counter	▪ Frequent cleaning ▪ Multi-dose
		Wixela inhub^®^	Manually slide a lever with the thumb, producing an audible click when a dose is ready	▪ Inexpensive	▪ Multi-dose
	Fluticasone + vilanterol	Breo Ellipta^®^	Slide down the cover until audible click and drop in dose counter	▪ Fewer steps to load a dose ▪ Dose counter ▪ Single daily dosing ▪ No routine cleaning required	▪ Long acting ▪ Cannot be used as a rescue inhaler
LABA/LAMA	Indacaterol + glycopyrrolate	Neohaler^®^	Capsule inserted into inhaler, pierced by pressing two side buttons, then inhale	▪ Single daily dosing	▪ Cumbersome drug-loading process

**Table 4:** A comparison of the advantages and disadvantages of using different types of nebulizers. Abbrev. AJN: air jet nebulizer.

Nebulizer	Advantages	Disadvantages
Air jet nebulizer	▪ Cost-effective ▪ Easy to use ▪ Can deliver formulations that cannot be delivered as pMDI or DPI ▪ More advanced devices are available to improve efficiency of drug delivery	▪ Inefficient delivery of drug ▪ Noisy ▪ Bulky ▪ Difficult to clean ▪ Requires external power source (separate compressor) and additional tubing ▪ Requires specific flow rates for operation ▪ Has large residual volume
Ultrasonic nebulizers	▪ Easy to use ▪ Quiet ▪ Efficient delivery of drug compared to AJNs ▪ No separate air compressor required	▪ Expensive ▪ Has large residual volume ▪ Cannot be used with viscous solutions ▪ Can degrade heat-sensitive material
Vibrating mesh nebulizers	▪ Easy to use ▪ Fast ▪ Quiet ▪ Portable ▪ Self-contained power source ▪ Efficient delivery of drug ▪ Minimal residual volume ▪ Adjustable flow rates to optimize drug delivery	▪ Expensive ▪ Difficult to clean ▪ Cannot aerosolize viscous solutions or solutions that crystalize upon drying
Smart nebulizers	▪ Provides feedback for patient ▪ Can accurately monitor delivered dose ▪ Adjusts to variable breathing pattern	▪ Expensive ▪ Largely untested in clinical trials

## Data Availability

No new data were created or analyzed in this review. Data sharing is not applicable to this article.
